# Histatin 8 Interactions with Copper, Zinc, and Nickel Ions, and Its Antimicrobial Profile in Relation to Histatin 5

**DOI:** 10.3390/molecules31010110

**Published:** 2025-12-28

**Authors:** Justyna Sokołowska, Joanna Słowik, Katarzyna Zamłyńska, Jolanta Kutkowska, Paweł Lenartowicz, Danuta Witkowska

**Affiliations:** 1 Institute of Health Sciences, University of Opole, Katowicka 68, 45-060 Opole, Poland; 2 Department of Genetics and Microbiology, Institute of Biological Sciences, Maria Curie-Skłodowska University, Akademicka Str. 19, 20-033 Lublin, Polandjolanta.kutkowska@mail.umcs.pl (J.K.); 3 Institute of Chemistry, University of Opole, Oleska 48, 45-052 Opole, Poland

**Keywords:** histatin 5, histatin 8, copper (II) complexes, isothermal titration calorimetry (ITC), binding mechanism, antimicrobial activity

## Abstract

Histatins are histidine-rich antimicrobial peptides present in human saliva, with histatin 5 (Hst5) demonstrating the most potent antifungal activity. Previous studies have linked the antifungal properties of histatins, particularly those against *Candida* species, to their ability to bind metal ions such as Cu(II) and Zn(II). While the antimicrobial activity of some histatins is well established, the impact of metal ion coordination on this activity remains an area of ongoing investigation. This study focuses on histatin 8 (Hst8), a less-explored member of the histatin family, and compares its metal-binding and antimicrobial properties to those of Hst5. Using isothermal titration microcalorimetry (ITC), we examined the interactions of Hst8 with Cu(II), Zn(II), and Ni(II) ions and evaluated its antimicrobial activity against *Escherichia coli*, *Staphylococcus aureus* and two *Candida albicans* strains. Our findings revealed significant differences in copper and zinc binding between Hst5 and Hst8, with both peptides exhibiting distinct antifungal profiles. Interestingly, it has been shown that copper ions bind to Hst5 in a distinctly different manner than to Hst8. Hst5 exhibits two binding sites with dissociation constants (K_DITC_) of 0.2 µM and 14.8 µM, whereas Hst8 has only one set of binding sites with a K_DITC_ of 12.3 µM. These results highlight the potential role of metal ion coordination in modulating the antimicrobial efficacy of histatins, providing further insight into their therapeutic potential.

## 1. Introduction

The human microbiome is a complex and dynamic system in which even minor disruptions of homeostasis can initiate a cascade of deleterious processes. Microorganisms interact not only with one another but also with host proteins, diverse biomolecules, and essential metal ions, all of which must be taken into account in such analyses. Commensal microorganisms that normally reside in the human body can become major threats when the host is weakened. In the context of rising antibiotic resistance, there is increasing interest in harnessing and enhancing endogenous components of the human body to act as effective allies in this ongoing struggle [1,2].

Human saliva and the gastrointestinal tract contain a wide array of compounds with antimicrobial properties. While some of these compounds have been extensively characterized, others continue to be discovered. Saliva, in particular, comprises diverse groups of antimicrobial peptides and proteins, including mucins, proline-rich proteins (PRPs), cystatins, statherins, cathelicidins, and low-molecular-weight histatins. Bioactive peptides and proteins found in the human body have attracted considerable interest as potential sources of novel antimicrobial therapeutics, owing to their low cytotoxicity and minimal side effects [3].

Several well-characterized antimicrobial peptides (AMPs) serve as robust templates for structural modifications aimed at enhancing potency or stability. However, it should be noted that engineering AMPs to increase activity against specific pathogens can sometimes lead to unpredictable or undesirable effects [4].

*In vitro* studies have confirmed the antifungal activity of histatins (1, 3, and 5) against various pathogenic fungi, particularly those from the genera *Candida*, *Cryptococcus*, and *Aspergillus* [5]. Moreover, in patients with HIV with opportunistic *Candida* infections in the oral cavity, reduced salivary histatin levels have been observed, suggesting that their primary function may be to prevent oral candidiasis [6]. Histatins are found in human saliva at concentrations ranging from 50 to 450 µM [3].

Many known histatins feature an NH_2_-XXH motif, recognized as the ATCUN motif (Amino-Terminal Cu(II) and Ni(II) binding site), which binds copper(II) and nickel(II) ions. In addition, they contain the Zn(II)-binding HEXXH motif, as well as polyhistidine-rich regions. The name of these compounds reflects their high histidine content in the primary structure. Studies have demonstrated that certain metal ions, particularly copper(II), can enhance the antimicrobial activity of peptides, including histatin 5 (Hst5) [7]. Cu(II) binding may increase Hst5 stability by protecting it from degradation by fungal proteases [8]. Some studies indicate that Zn(II) complexes with Hst5 exhibit greater antifungal properties than the peptide alone [9], whereas others suggest that Zn(II) can reduce Hst5 activity. It has been proposed that zinc exerts a concentration-dependent effect on the biological activity of Hst5 [10].

**Figure 1:**
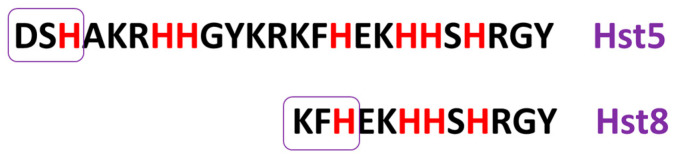
Sequences of the tested peptides with corresponding abbreviations (shown on the right) used in the publication. The ATCUN-like motifs are highlighted with frames, whereas the histidine residues, the most likely binding sites for the studied divalent metal ions at physiological pH, are marked in red.

Among the histatin family members, histatin 5 has the most potent antifungal activity identified to date, establishing it as a key molecule of interest in antimicrobial peptide research. Hst5 is a 24-amino-acid proteolytic fragment derived from histatin 3. Its amino acid sequence is presented in Figure 1. Produced and secreted by the sublingual, parotid, and submandibular glands, Hst5 is a natural and essential component of human saliva, where it plays a critical role in oral host defense mechanisms. Human saliva has also been shown to contain various metal ions, including zinc, copper, iron, nickel, and manganese, with Zn(II) being the most abundant [9].

Despite extensive research, the antimicrobial mechanism of histatins remains incompletely understood. In particular, histatin 8 (Hst8) has been poorly characterized with respect to both its antimicrobial properties and its interactions with metal ions. It is reasonable to hypothesize that, like Hst5, Hst8 may also interact with copper ions through its NH_2_-XXH motif or histidine residues. The presence of histidine-rich sequences in histatins suggests broader potential for metal binding, which could influence their biological activity and stability. Since Hst8 has not been extensively studied, several key research questions remain open. One of the primary uncertainties is how its interaction with metal ions differs from that of Hst5 in a physiological environment. Additionally, it is unclear whether these interactions influence the antimicrobial activity of Hst8 and whether Hst8 has antifungal or antibacterial properties comparable to those of Hst5.

Our previous research has shown that histidine-rich peptides can bind multiple metal ions, including Cu(II), Ni(II), and Zn(II), through the imidazole side chains of histidine residues and other peptide functional groups [11,12,13]. Given this, and despite earlier reports suggesting that Hst5 binds copper at a 1:1 ratio [3], we sought to verify this hypothesis. To achieve this goal, we conducted studies at near-physiological pH using the isothermal titration microcalorimetry (ITC) technique.

In this study, we also investigated the interactions of Hst8 with copper, zinc, and nickel divalent ions, as well as its antimicrobial activity against two bacterial and two *Candida* strains, in direct comparison to Hst5. To ensure a robust and reliable evaluation, all experiments were conducted under identical conditions for both peptides, enabling a comprehensive assessment of their metal coordination properties and antimicrobial potential. The sequences of both tested peptides are presented in Figure 1.

## 2. Results

### 2.1. ITC and UV-Vis Spectroscopy Binding Experiments

**Figure 2:**
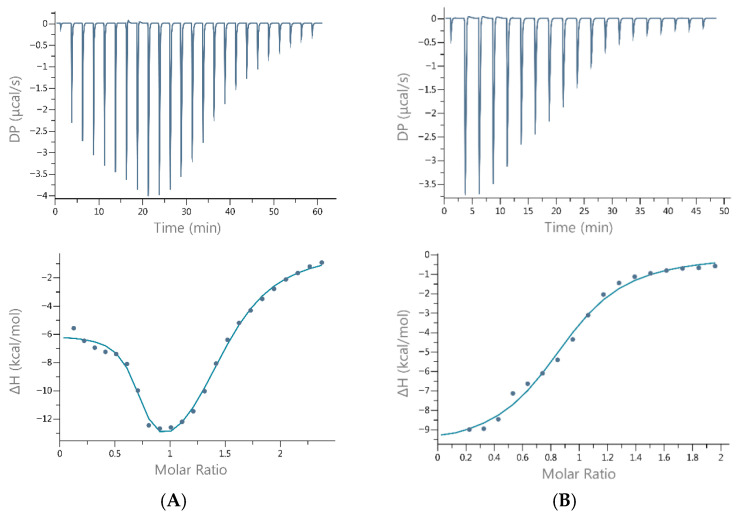
Representative ITC binding data for the titration of Cu(II) into (**A**) histatin 5 and (**B**) histatin 8 in 20 mM MOPSO, pH 7.0, at 25 °C. The top panels show the differential power signals (thermograms) recorded for each injection, whereas the bottom panels present the binding isotherm constructed by fitting the points corresponding to the integration of the peaks in the thermograms (representing the heat released during each injection) as a function of the Cu(II)/Hst molar ratio.

Isothermal titration calorimetry (ITC) experiments were conducted to assess the strength of copper(II) interactions with Hst5 and Hst8, as well as to determine the binding stoichiometry and the enthalpy and entropy changes. Given the condition-sensitive nature of ITC measurements, all experiments were conducted under uniform experimental conditions (pH, buffer, temperature) to guarantee reproducible and comparable results. The results are presented in Table 1 and Figure 2. Additionally, measurements with Ni(II) and Zn(II) ions were performed (see Supplementary Table S1 and Figures S1 and S2). Each assay was repeated multiple times, and the results represent the averages of the two best-fitting measurements. The apparent parameters were obtained directly from the ITC experiment by fitting the binding isotherms using nonlinear least-squares analysis, which is based on a model that assumes a single set of identical binding sites [14]. However, for Cu(II)-Hst5 complexation, fitting to a single set of identical binding sites was not feasible, and a two-site binding model provided a significantly better fit [15].

The results for the interaction of Cu(II) with histatin 5 revealed two binding sites: one with high affinity (0.2 µM) and another with moderate affinity (~15 µM). In contrast, the interaction between Cu(II) and histatin 8 resulted in a single binding site on the peptide with moderate affinity for Cu (II) (~12.3 µM). All interactions were enthalpically driven (Table 1).

In the case of Zn(II) and Ni(II) interactions with Hst5 and Hst8, significantly weaker affinities were observed (K_DITC_ ranging from 110 to 238 µM). These interactions were also enthalpically driven, with significant entropic contributions detected for Zn(II) binding to Hst8. Both histatin titrations with Ni(II) at the same pH revealed that only half of the expected binding sites were engaged in the interaction (N ≈ 0.5). In contrast, zinc interactions with the studied histatins exhibited distinct differences in stoichiometry and the underlying driving forces of the reaction. All complexes are characterized by comparable Gibbs free energy changes (ΔG), which were calculated from the dissociation constants determined by ITC according to the thermodynamic relationship ΔG = RT ln K_D_.

UV-Vis spectroscopy titration of the peptides with the metal cation was performed up to a Cu:Hst ratio of 1.6:1, with scans recorded after each addition of 0.2 equivalents of Cu(II). For the Cu-Hst5 complex, the maximum absorbance was observed at 520 nm for a 1:1 ratio, shifting slightly to 530 nm at a 1.6:1 ratio (Figure S3). These maxima indicate the formation of square-planar Cu-Hst5 complexes, in which each Cu(II) ion is coordinated by four nitrogen atoms: one from the N-terminal amino group, two from amide groups, and one from the imidazole side chain of His3 [8,12]. During the formation of the Cu(II)-Hst8 complex, a maximum absorbance in the d-d transition region was observed at 515 nm at a Cu(II):Hst8 ratio of 1:1 (Figure S4, Table S2). This absorption is characteristic of Cu(II) coordination to four nitrogen donor atoms, and is consistent with previous observations for the Cu(II)–Hst5 complex [8,12]. The assignment of the metal-binding sites was not based on the concentration-dependent spectra alone, but on a combination of diagnostic spectroscopic features, thermodynamic parameters, and comparison with established coordination motifs. The d–d transition maxima observed for Cu(II) (515–520 nm) and Ni(II) (~420 nm) fall within narrow, well-defined ranges characteristic of square-planar ATCUN-type 4N coordination, providing a clear spectroscopic signature of N-terminal binding. The stepwise thermodynamic constants further distinguish the high-affinity ATCUN site from weaker secondary sites, consistent with reports for histatin-rich peptides [12,13].

Since the affinity of zinc (II) ions for Hst5 and Hst8 was weak (K_DITC_ values of 238 and 195 µM, respectively), we focused on investigating whether Cu(II) ions enhance the antimicrobial activity of these histatins.

### 2.2. MIC Determination of Histatins and Their Cu(II) Complexes

The tested histatins and their copper complexes showed moderate antibacterial activity (Table 2). Hst5 showed anti-yeast activity at a concentration of 82 µM, but did not inhibit bacterial growth (MIC > 165 µM). Compared with the native preparations, the complexes of Hst5 with Cu(II) ions had greater antibacterial activity. The MIC value decreased two-fold for *E. coli* ATCC 25922, reaching 82 µM, and for *S. aureus* ATCC 25923, it stayed at the level of 165 µM. No greater activity of Hst5 copper complexes against the tested *C. albicans* strains was observed.

Hst8 revealed the best antibacterial efficacy against *E. coli* ATCC 25922 at a concentration of 160 µM, and was less active against the other microorganisms tested, for which the MIC values were 320 µM. In the case of the Hst8 copper complex, a decrease in the MIC value (from 320 to 160 µM) was observed only for the *C. albicans* ATCC 10231 strain. Ampicillin and fluconazole were used as antibacterial and antifungal positive controls, respectively. Ampicillin inhibited the growth of *E. coli* ATCC 25922 and *S. aureus* ATCC 25923 at concentrations of 5.7 and 2.85 µM, while fluconazole was effective against *C. albicans* ATCC 90028 (MIC of 13 µM) and *C. albicans* ATCC 10231 (MIC of 26 µM).

### 2.3. Microorganism Growth Curve Assay

**Figure 3:**
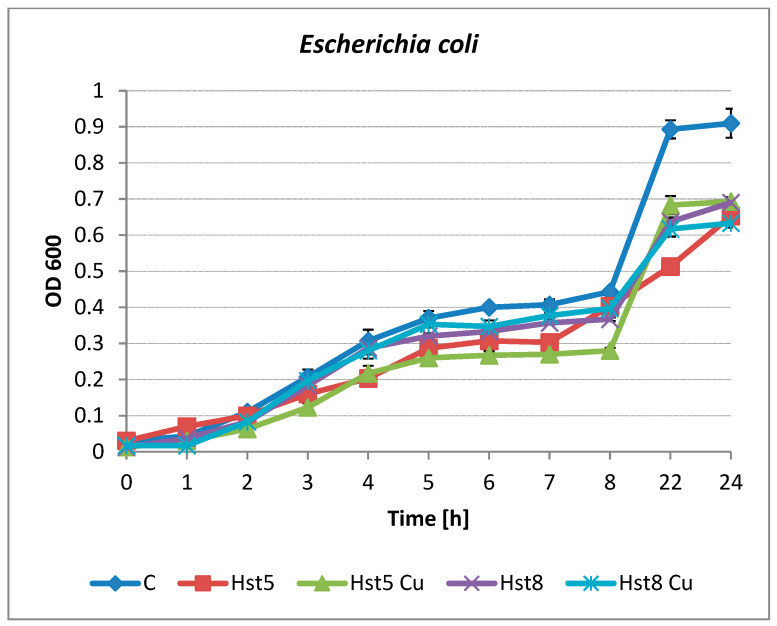
Growth of *E. coli* ATCC 25922 in the presence of Hst5 and Hst8 and copper complexes at a concentration of 200 µg/mL. C—control (peptide-free microbial culture), Hst5—histatin 5, Hst5 Cu—histatin 5 Cu complex, Hst8—histatin 8, Hst8 Cu—histatin 8 Cu complex.

Kinetic growth inhibition analyses were carried out for *E. coli* ATCC 25922 and *C. albicans* ATCC 90028. Both tested histatins, Hst8 and Hst5, as well as their Cu(II) complexes at a concentration of 200 µg/mL, inhibited the growth of *E. coli*. Changes were noticeable as early as the 3rd hour of culture in the case of Hst5 and its complex (inhibition of 23 and 40%). This effect was observed in the following hours of incubation, and the 24 h growth inhibition rates were 29 and 24%, respectively (Figure 3). Hst8 and its copper complex showed antibacterial activity after 6 h of *E. coli* growth (inhibitions of 17 and 14%); the highest percentage of growth inhibition (30–31%) was observed after 22 h of incubation.

**Figure 4:**
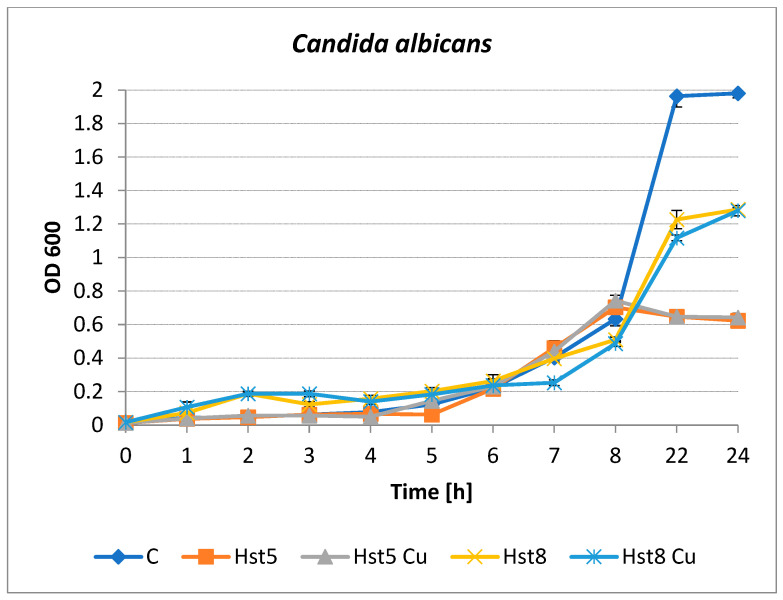
Growth of *C. albicans* ATCC 90028 in the presence of histatins 5 and 8 and copper complexes at a concentration of 200 µg/mL. C—control (peptide-free microbial culture), Hst5—histatin 5, Hst5 Cu—histatin 5 and Cu complex, Hst8—histatin 8, Hst8 Cu—histatin 8 and Cu complex.

Inhibition of the growth of *C. albicans* by histatins was observed after 4–8 h of incubation (Figure 4). Hst5 and its Cu(II) complex were the most active species at longer incubation times. After 22 and 24 h of incubation, yeast growth inhibition of approximately 70% was observed for both preparations. The differences in yeast growth were not so clear after 22–24 h for Hst8 and the Cu(II) complex. Hst8 was found to inhibit the growth of *C. albicans* to a small but statistically significant extent after 5 h of culturing, after which antimicrobial activity was not detected (*p* < 0.05). The copper complex of Hst8 showed an inhibitory effect on the growth of *C. albicans* after 6 h compared to the culture containing Hst8 and the control (*p* < 0.05). The percentage of microbial growth inhibition was calculated using the following formula: % inhibition = [(OD control − OD sample)/OD control] × 100, where OD control = the optical density of the control microbial cultures without peptides; OD sample = the optical density of the cultures after incubation with histatins.

No statistically significant differences (*p* > 0.05) were found between the culture of *E. coli* in Mueller-Hinton medium (control) and the culture of the strain with the addition of copper ions (control + Cu(II)).

## 3. Discussion

### 3.1. Metal Interactions of Histatins

Histatin 5, like other polyhistidyl peptides, has been extensively studied. However, research on histatin 8 remains limited. We found only one study investigating its interactions with metal ions and the antimicrobial properties of its complexes [16].

In this work, we employed isothermal titration microcalorimetry (ITC) to study Hst5 and Hst8 interactions with Cu(II), Zn(II) and Ni(II) ions, which provides simultaneous insights into the stoichiometry, binding affinity, and thermodynamic driving forces of these interactions. Unlike potentiometric methods, ITC measurements are conducted in aqueous buffer under constant pH conditions (pH 7.0 here), closely mimicking the physiological environment of human saliva. This makes ITC a powerful tool for investigating interactions between natural products or their analogs (e.g., histatins) and metal ions, offering deeper insights into their fundamental binding mechanisms. As a complementary technique, we employed UV-Vis spectroscopy to elucidate the geometry of the Cu(II) and Ni(II) complexes with both histatins.

There are discrepancies in the reported results of Cu(II) binding to Hst5. Some studies suggest that Hst5 has only a single binding site for copper ions [16], whereas others indicate the presence of two distinct binding sites [8]. Brewer and Lajoie, using electrospray ionization mass spectrometry (ESI-MS), demonstrated the presence of multiple binding sites for Cu(II) and Ni(II) ions in Hst5, whereas only a single binding site was identified for Zn(II) [17]. On the other hand, Gusman et al. suggested that histatin 5 possesses one binding site with high selectivity for zinc, and two sites with low affinity for these metal ions [18]. Similarly, they demonstrated the presence of three binding sites on Hst5 for Cu(II) ions. Norris et al. demonstrated that the higher relative affinity of Hst5 for Zn(II) likely facilitates dimerization [9]. All studies agree that zinc ions bind to Hst5 with lower affinity compared to nickel and copper ions what is also shown by our results. NMR studies have shown that the preferred binding site for Zn(II) is -^3^H-E-X-X-H^7^- [16,19]. Cragnell and coworkers, using multiple techniques, revealed that Zn(II) binding to Hst5 induces oligomer formation as the zinc ion concentration increases. Dimers are formed through imidazole-mediated binding of two Hst5 chains, maintaining a 1:1 (2:2) stoichiometry, while allowing for multiple distinct coordination modes [20]. Most studies on Cu-Hst5 complexes indicate that copper binding enhances histatin antimicrobial activity, whereas only a few suggest a similar effect for zinc. It has been proposed that the formation of a Cu(II)-histatin complex is a prerequisite for the oxidative activity of Hst5 [21]. The ability of histatins to bind metal ions—not only copper—may be a key property with diverse applications beyond their antimicrobial activity, such as sequestering and detoxifying toxic metals such as Ni(II).

Bal and colleagues utilized potentiometry and spectrophotometry to investigate Ni(II) binding to Hst5, demonstrating strong coordination of one Ni(II) ion at its N-terminal site and weaker binding at the C-terminal site [22]. The binding geometry corresponds to a square-planar arrangement, consistent with previously reported structures and with the Cu(II) coordination observed at the N-terminal region of Hst5 and Hst8 in our study.

In our studies with histatin 5 and its shorter analog, histatin 8, we identified two binding sites on Hst5 exclusively for copper ions. Nickel and zinc ions bind to Hst5 and Hst8 with approximately ten times weaker affinity. However, because of interactions between the metal ions and the buffer, direct quantitative comparison between Hst5 and Hst8 across different metals is not possible; only the relative differences in their interactions with each individual metal can be reliably assessed, as was done in the present study.

Interestingly, copper ions bind to Hst5 in a distinctly different manner than to Hst8. Hst5 exhibits two binding sites with dissociation constants (K_DITC_) of 0.2 µM and 14.8 µM, whereas Hst8 has only one set of binding sites with a K_DITC_ of 12.3 µM (Table 1). This difference is consistent with the sequence of Hst5, which, in addition to the ATCUN motif, contains two dihistidyl motifs and two additional histidine residues—potential anchoring sites. Hst8 has a single dihistidyl site, but its proximity to the ATCUN motif likely prevents the binding of a second copper atom or renders that interaction too weak to be detected under these measurement conditions. At pH 7.0, the differences in nickel binding between Hst5 and Hst8 are minimal, with Hst5 showing a slightly greater affinity for nickel ions (Table S1). Our studies show that Hst5 and Hst8 bind Zn(II) via distinct mechanisms. Distinct mechanisms of both peptides for Zn(II) and Cu(II) have also been demonstrated by Dzien et al. In their work, the authors showed that, for Zn(II) complexes, the most stable species across the entire pH range is formed with histatin 5, whereas for Cu(II) complexes, histatin 8 exhibits greater thermodynamic stability throughout the tested pH range. It should be noted that these measurements were performed under different experimental conditions, and the thermodynamic equilibrium was established differently than in the present study [16]. In the present study, both interactions with zinc were shown to be relatively weak; Hst5 binding was enthalpically driven, whereas Hst8 binding was predominantly entropy-driven (Table S1). Despite similar ΔG values, differences in ΔH, entropic contributions, and stoichiometry highlight distinct coordination behaviors. For Hst5, N ≈ 0.5 suggests metal-bridging between two peptide molecules or peptide oligomerization, consistent with a strongly negative ΔH (−6.8 kcal·mol^−1^) and unfavorable entropy. In contrast, Hst8 exhibits N ≈ 1, near-zero ΔH (−0.6 kcal·mol^−1^), and favorable entropic contribution (−TΔS ≈ −4.5 kcal·mol^−1^), indicating entropy-driven binding likely mediated by solvent release, hydrophobic effects, or changes in aggregation state. The low stoichiometry for Hst5 may also reflect incomplete histidine deprotonation at pH 7.0 or metal-induced oligomerization, as previously reported by Cragnell et al. [20]. The proposed coordination mode involving inter-peptide imidazole interactions, which can lead to oligomer formation, is shown in Figure S5. The structure shown in Figure S5 represents a schematic illustration of a probable dimerization process inferred from the ITC-derived stoichiometry (*n* ≈ 0.5), rather than a directly determined structural model. This interpretation is supported by previous studies demonstrating histidine-mediated peptide oligomerization during interactions with divalent metal ions [23,24].

The number of metal-binding sites detected for histatin 5 varies among different studies, which is likely related to methodological and environmental factors (Table 3). A 1:1 Cu(II):Hst5 stoichiometry has been predominantly reported in spectroscopic studies (UV–Vis, CD, NMR), which are typically conducted at low Cu(II) concentrations and in weakly or non-coordinating buffers. Under such conditions, these techniques primarily probe the dominant, high-affinity ATCUN site, which is widely recognized as the principal Cu(II) anchoring motif in histatin 5. In contrast, higher apparent stoichiometries (2:1 or ≥2:1 for Cu(II):Hst5) have been reported using methods such as ESI-MS, potentiometry, and ITC, particularly at elevated Cu(II):peptide ratios, at pH ≥ 7. Techniques such as ITC are sensitive to all enthalpically active events and can therefore reveal both high- and low-affinity binding processes, while spectroscopic methods often capture only the dominant coordination site. In addition, both buffer composition and pH can significantly influence metal—peptide equilibria. Some buffers, such as MOPSO or HEPES, can weakly coordinate Cu(II) ions, altering their availability and possibly stabilizing secondary binding events. Moreover, the protonation state of histidine residues changes with pH, which can affect the accessibility and affinity of alternative metal-binding sites. Importantly, an apparent stoichiometry of *n* ≈ 2 observed by ITC does not necessarily indicate two equivalent and independent Cu(II) binding sites; rather, it is consistent with the coexistence of a high-affinity ATCUN site and a weaker, context-dependent interaction, likely involving His–His motifs and/or inter-peptide coordination. From this perspective, the present ITC data are not contradictory to earlier spectroscopic reports but instead provide a more comprehensive thermodynamic description of Cu(II) binding to Hst5. Notably, previous studies focused exclusively on Hst5 and employed disparate buffers, pH conditions, and analytical techniques, precluding a direct assessment of whether the reported differences in Cu(II) stoichiometry arise from intrinsic sequence features or from experimental variability. By directly comparing Hst5 with its truncated analog Hst8 under strictly identical experimental conditions, the present study addresses this limitation and demonstrates that the additional Cu(II) binding event is sequence-dependent and absent in Hst8, thereby clarifying the molecular origin of the higher stoichiometries reported for Hst5.

Indeed, this approach clearly shows that, under physiological conditions, Hst8 exhibits weaker copper-chelating ability than Hst5, and that the coordination modes of these two peptides differ.

### 3.2. Biological Activity

The mechanism of action of antimicrobial peptides assumes their interaction with the pathogens’ membranes. AMPs can bind to microorganisms directly through electrostatic interactions with anionic groups on bacterial cell membranes and then penetrate the bacterial cell membrane, resulting in their death. Some cell-penetrating peptides exhibit strong antibacterial activity across the cell membrane without disturbing the intracellular interaction mechanisms [25]. The mechanisms of action of Hst5 on *C. albicans* fungal cells suggest the involvement of many intracellular targets: nonlytic leakage of ATP and K^+^ ions, mitochondrial damage, and the generation of oxidative stress. Hst5 has been identified as the most potent histatin in inhibiting the growth of both yeast and filamentous forms of *Candida* [26].

Due to the nonspecific mechanisms of AMP activity, microorganisms have not developed resistance mechanisms, unlike many currently used antibiotics. Therefore, these compounds are among the most promising drug candidates for the development of new antibiotics [25].

The results of our studies confirmed the activity of Hst5 against *C. albicans* strains, whereas Hst8 showed lower anti-yeast activity (MICs of 320 µM). The Hst8 copper complex showed greater activity (twofold lower MIC value) against the *C. albicans* ATCC 10231 strain, compared to the native peptide. Both histatins and copper complexes at concentrations below the MIC apparently inhibited *C. albicans* growth after 22–24 h of incubation compared with the control, but Hst5 and its complex with Cu had greater activity.

Although our tests did not show any inhibition of the growth of the bacterial strains used, an increase in the antibacterial activity of the Hst5-copper complex was observed. Hst8 had the best antibacterial efficacy against *E. coli* ATCC 25922, but it also inhibited the growth of *S. aureus* (MICs of 160µM and 320 µM, respectively). No increase in antibacterial activity was demonstrated after Hst8 was complexed with Cu(II) ions.

Du et al. [26] described that Hst5 possesses high bactericidal activity against some pathogens that cause nosocomial infections. The MIC values for Gram-negative bacteria (*Acinetobacter baumanii*, *Pseudomonas aeruginosa*, and *Enterobacter* sp.) ranged from 38 to 90 µM, which corresponds to concentrations in the range of 115.3–273 µg/mL Hst5.

The results obtained by Matheson et al. [27] demonstrated a narrow spectrum of Hst8 anti-yeast activity against *C. albicans* and *C. tropicalis* (MICs of 1 and 5 mg/mL, respectively). However, methicillin-resistant *Staphylococcus aureus* (MRSA) and *E. coli* were not sensitive to this peptide. Other authors reported that Hst8 was active against drug-resistant strains of *A. baumannii* (MIC 32 µg/mL) [28]. The differences in the thermodynamic parameters and metal-binding mechanisms observed for Hst5 and Hst8 can have important biological implications in the context of oral innate immunity. The higher affinity and multiple Cu(II)-binding sites of Hst5 suggest a greater capacity to modulate copper availability and potentially promote copper-mediated oxidative stress, which may preferentially enhance antibacterial activity. In contrast, the weaker and single-site Cu(II) binding of Hst8, together with its different thermodynamic signature, appears to favor antifungal activity, possibly through altered interactions with fungal cell wall components rather than metal sequestration. While Hst5 shows only moderate antifungal activity, it consistently displays higher activity against *C. albicans* compared to Hst8, indicating a relative advantage rather than strong intrinsic potency.

## 4. Materials and Methods

### 4.1. Isothermal Titration Calorimetry (ITC)

ITC measurements were conducted at 25 °C via a MicroCal PEAQ isothermal titration calorimeter (Malvern Panalytical, Malvern, UK). All reagents were obtained from Sigma-Aldrich (Buchs, Switzerland) and were of >99% purity. The peptides (ordered from KareBay Biochem, Monmouth Junction, NJ, USA) were dissolved directly in a 20 mM MOPSO (2-hydroxy-3-morpholinopropanesulfonic acid) buffer, pH 7.0. Metal ion stock solutions were prepared in deionized water (maximum conductivity of 0.05 μS/cm) at low pH (~2) in glass bottles.

After the instrument was stabilized at 25 °C, titrations were performed using 40 μL of metal ion solutions (2 mM for Cu(II) and 4 mM for Ni(II) and Zn(II) ions) to titrate 200 μL of histatin solutions, with an initial peptide concentration ten times lower than that of the metal ions. The titration consisted of 19 successive injections, with intervals of 150–180 s between each injection [29]. Each assay was repeated a few times to ensure reproducibility.

A background titration was subtracted from the results to account for the heat of dilution. The stirring rate was set at 750 rpm throughout the experiments, and the reference cell was filled with demineralized water. Data were processed using MicroCal PEAQ-ITC Analysis Software (v1.20). An initial 0.4 μL injection was discarded from each data set to eliminate the effect of titrant diffusion across the syringe tip during the equilibration process. The ITC results are reported in terms of K_DITC_ (μM), ΔH_ITC_ (kcal/mol), and N_ITC_. K_DITC_ (μM) represents the equilibrium dissociation constant obtained from ITC analysis under specific conditions, ΔH_ITC_ (kcal/mol) represents the binding enthalpy, and N_ITC_ corresponds to the number of binding sites per macromolecule.

### 4.2. UV-Vis Spectroscopy

UV-Vis absorption spectra were recorded via a Cintra 3030 spectrophotometer (GBC Company, Dandenong, VIC, Australia) over the wavelength range of 200–800 nm. Quartz cuvettes with a 1 cm optical path length (BioSens, Warszawa, Poland) were used for all measurements. Peptide and Cu(II) solutions were prepared in MOPSO buffer (20 mM, pH 7.0). The peptide concentration was maintained at 1 mM. The spectra were collected at 25 °C following the incremental addition of Cu(II) ions to the peptide solution. The titration points included 0, 0.2, 0.4, 0.6, 0.8, 1.0, 1.2, 1.4, and 1.6 equivalents of Cu(II) relative to the peptide concentration. Each spectrum was recorded after allowing the solution to equilibrate for 5 min to ensure complex formation.

### 4.3. Antimicrobial Activity Assay

#### 4.3.1. Determination of the Minimum Inhibitory Concentration (MIC)

As the studied histatins formed the most thermodynamically stable complexes with copper(II) ions, these complexes were selected for subsequent antimicrobial investigations.

The antimicrobial activity of histatins 5 and 8 and their complexes with Cu(II) was determined via a microdilution method in a 96-well plate (Biologix Europe GmbH, Niederzier, Germany) against the bacterial strains *Escherichia coli* ATCC 25922 and *Staphylococcus aureus* ATCC 25923, and the two yeast strains *Candida albicans* ATCC 90028 and *C. albicans* ATCC 10231 derived from the American Type Culture Collection (Manassas, VA, USA) according to the Clinical and Laboratory Standards Institute guidelines [30]. The experiments were carried out in liquid medium Muller Hinton Broth (MHB) or Sabouraud Dextrose Broth (SDB) (BioMaxima S.A., Lublin, Poland) for bacteria and yeast, respectively.

Histatins were dissolved in sterile saline (0.9% NaCl) to obtain a concentration of 1 mg/mL, and then a series of twofold dilutions was made in broth appropriate for the tested strains at a concentration ranging from 500 to 31 μg/mL. Additionally, saline with Cu(II) ions in a concentration of 1 μM was used as a control for complexes with Cu(II). Ampicillin and fluconazole were used as positive controls for *E. coli* (at concentrations ranging from 1 to 16 μg/mL) and *C. albicans* (at concentrations ranging from 0.5 to 8 μg/mL), respectively. Peptide-Cu(II) complexes were prepared at a 1:1 molar ratio (peptide:Cu) and incubated for 30 min at room temperature prior to the assays. A broth-free medium was used as a control for microbial growth.

The suspension of tested strains was adjusted to the 0.5 McFarland standard and diluted in appropriate medium to obtain a final density of 5 × 10^6^ CFU/mL. The microplates were incubated at 37 °C for 18 h, and microbial growth was read spectrophotometrically via a microplate reader ASYS UVM 340 (Biochrom Ltd., Cambridge, UK). All experiments were performed in triplicate.

The minimum inhibitory concentration (MIC) values were defined as the lowest compound concentrations inhibiting visible in vitro microbial growth and were recalculated into micromolar units to express molar concentrations.

#### 4.3.2. Growth Curve Assay

The determination of the growth curves of *E. coli* ATCC 25922 and *C. albicans* ATCC 90028 in the presence of histatins and their complexes with Cu was performed by measuring the optical density of microbial cultures at 600 nm (OD600) against a control containing medium using an Implen OD600 DiluPhotometer (Implen GmbH, München, Germany). Cultures of the tested strains were carried out in a volume of 3 mL of MHB or SDB. Histatins at a concentration of 200 μg/mL (concentration lower than the MIC value) were used; the controls were cultures of microorganisms without histatins and additionally cultures with Cu(II) ions at a concentration of 5 μl/mL (1 μM). Microbial suspensions with a final density of 5 × 10^6^ CFU/mL were used. Measurements were carried out for 8 h at 1-h intervals, and then after 22 and 24 h.

All experiments were performed in triplicate. Statistical analysis was performed using Student’s *t*-test (Statistica 13.3 software). Data are presented as mean ± standard deviation (Mean ± SD), with *p* < 0.05 considered statistically significant.

## 5. Conclusions

Our study provides new insights into the metal-binding properties of histatin 5 and histatin 8, highlighting significant differences in their interactions with Cu(II) and Zn(II) ions. ITC measurements revealed that Hst5 possesses two distinct binding sites for copper ions, whereas Hst8 has only one, reflecting differences in their sequences and structural motifs. The observed variations in metal affinity and thermodynamic parameters suggest that these peptides interact with metal ions in unique ways, which may influence their biological functions.

Furthermore, our findings confirm the antifungal activity of Hst5 against *C. albicans*, with Hst8 displaying lower efficacy. Notably, complexation with copper increased the antifungal activity of Hst8, reducing the MIC value twofold. While neither peptide significantly inhibited bacterial growth, Hst8 showed the greatest antibacterial effect against *E. coli*. However, complexation with Cu(II) did not enhance its antibacterial properties.

These results suggest that metal coordination plays a crucial role in modulating the biological activity of histatins, particularly in antifungal applications. It should be noted that, in this form, neither the peptides nor their copper complexes can serve as antibacterial agents.

Further modifications of these peptides, guided by AI-based design, could enhance their antimicrobial activity and improve their stability, making them even more promising for therapeutic applications. Of course, in such applications one should remember about the synergistic effects of various proteins and peptides, as well as about interactions between microorganisms inhabiting a given area of the human body and other conditions, e.g., concomitant diseases.

## Figures and Tables

**Table 1:** Experimental (conditional) thermodynamic parameters for Cu(II) binding to Hst5 and Hst8, determined from ITC measurements in MOPSO buffer, 25 °C.

Ligand	$K_{DITC}\;[µM]$	ΔH_ITC_ [kcal/mol]	N_ITC_ [Sites]
Hst5	0.20 ± 0.05	−5.6 ± 0.4	0.60 ± 0.01
	14.8 ± 1.2	−14.0 ± 0.3	0.80 ± 0.03
Hst8	12.3 ± 3.1	−9.5 ± 0.6	0.90 ± 0.02

**Table 2:** Antimicrobial activity of histatins and complexes with Cu(II) ions presented as Minimum Inhibitory Concentration (MIC) values in µM.

	MIC (µM)			
	*E. coli* 25922	*S. aureus* 25923	*C. albicans* 90028	*C. albicans* 10231
Hst5	>165	>165	82	82
Hst5 + Cu(II)	82	>165	82	82
Hst8	160	320	320	320
Hst8 + Cu(II)	160	320	320	160
Ampicillin	5.7	2.85	-	-
Fluconazole	-	-	13	26

**Table 3:** Summary of the coordination characteristics of Cu(I)/(II), Zn(II), and Ni(II) ions with histatin 5 (Hst5), based on spectroscopic and thermodynamic studies.

Metal Ion	Experimental Method	Number of Binding Sites	Binding Strength/Key Findings	Representative References
Cu(II)	UV–Vis, EPR, CD, potentiometry, ITC, NMR, MS	1 site (N-terminal ATCUN motif: NH_2_–Asp–Ser–His–)	High affinity; log K ≈ 5–7 (K_D_ ≈ 10^−5^–10^−7^ M). The complex is square-planar, coordinated through the terminal amine, deprotonated amide nitrogens, and the imidazole of His.	[16,19]
Cu(I)/Cu(II)	Spectrophotometry, X-ray absorption spectroscopy, ITC	3 possible sites (ATCUN motif and 2 bis-His)	1 high-affinity Cu(II)-binding site K_D_ up to ∼8 pM, two weaker copper binding sites.	[8,18]
Zn(II)	ITC, UV–Vis, fluorescence spectroscopy, NMR, Potentiometry, MS	Up to 3 possible sites (main-HEKHH)	Moderate affinity; log K ≈ 4–5 (K_D_ ≈ 10^−4^–10^−5^ M). Binding is weaker than for Cu(II) and likely transient under physiological conditions.	[16,18,19]
Ni(II)	Potentiometry, UV–Vis, CD	1 main site (ATCUN-like motif), second much weaker site	log K ≈ 7.5–8.0 (K_D_ ≈ 10^−8^ M). The coordination geometry is similar to that of Cu(II), but with lower stability.	[22]
Cu(II), Ni(II), Zn(II)	ESI-MS, CD	Multiple binding sites for Cu(II) and Ni(II), and one for Zn(II)		[17]

## Data Availability

The raw data supporting the findings of this study are available from the corresponding author upon reasonable request.

## Supplementary Materials

The following supporting information can be downloaded at: https://www.mdpi.com/article/10.3390/molecules31010110/s1, Figure S1: Representative ITC binding data for the titration of Ni(II) into (A) histatin 5 and (B) histatin 8 and Zn (II) into (C) histatin 5 and (D) histatin 8, in 20 mM MOPSO, pH 7.0, at 25 °C. The top panels show the differential power signals (thermograms) recorded for each injection, whereas the bottom panels present the binding isotherm constructed by fitting the points corresponding to the integration of the peaks in the thermograms (representing the heat released during each injection) as a function of the metal/Hst molar ratio; Figure S2: Signature plots of the titration of Ni(II) into (A) histatin 5 and (B) histatin 8 and Zn (II) into (C) histatin 5 and (D) histatin 8, in 20 mM MOPSO, pH 7.0, at 25 °C; Figure S3: UV-ViS results of Cu(II)-Hst5 binding in 20 mM MOPSO, pH 7.0, at 25 °C; Figure S4: UV-ViS results of Cu(II)-Hst8 binding in 20 mM MOPSO, pH 7.0, at 25 °C; Figure S5: Proposed coordination mode of M(II) complexes with the interpeptide imidazoles, that can lead to oligomer formation; Table S1: Results of ITC-based interaction studies of the examined histatins with Ni(II) and Zn(II) ions, performed in MOPSO buffer at 25 °C; Table S2: Maximum wavelength of absorbance in the d-d transition region for Cu(II) and Ni(II) complexes with Hst5 and Hst8 at a 1:1 ratio, measured in MOPSO buffer, at pH 7.0.
