# Supplementary material for: Histatin 8 Interactions with Copper, Zinc, and Nickel Ions, and Its Antimicrobial Profile in Relation to Histatin 5
Source: Molecules. 2025 Dec 28;31(1):110. doi: 10.3390/molecules31010110 (PMC12787115; doi:10.3390/molecules31010110)
Supplement: Supplementary file 1 [file molecules-31-00110-s001.zip › molecules-4030757-supplementary.pdf]

Supplementary material

Histatin 8 interactions with copper, zinc, and nickel ions, and its antimicrobial profile in relation to Histatin 5

Justyna Sokołowska[1], Joanna Słowik[1], Katarzyna Zamłyńska[2], Jolanta Kutkowska[2], and Paweł Lenartowicz[3], Danuta Witkowska[1*]

Table S1. Results of ITC-based interaction studies of the examined histatins with Ni(II) and Zn(II) ions, performed in MOPSO buffer at 25°C.

	K_{DITC} [μM]	ΔH [kcal/mol]	- TΔS [kcal/mol]	N [sites]
**Ni(II)-Hst5**	110 ± 6	-11 ± 0.3	5.0	0.5 ± 0.1
**Ni(II)-Hst8**	203 ± 31	-7.5 ± 1	2.5	0.4 ± 0.0
**Zn(II)-Hst5**	238 ± 28	-7 ± 1	1.8	0.5 ± 0.1
**Zn(II)-Hst8**	195 ± 72	-0.6 ± 0.1	- 4.5	1.1 ± 0.1

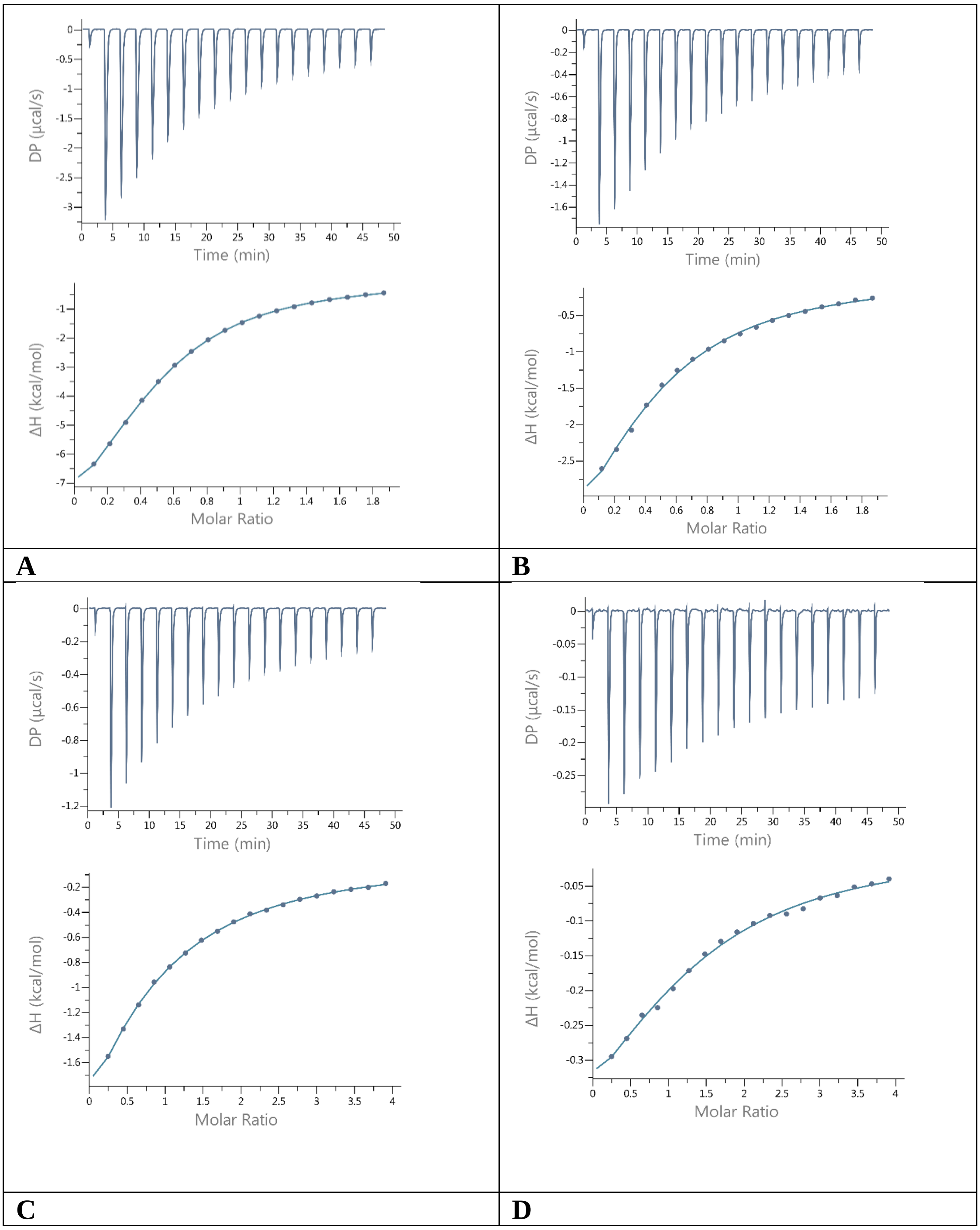

Figure S1. Representative ITC binding data for the titration of Ni(II) into (A) Histatin 5 and (B) Histatin 8 and Zn (II) into (C) Histatin 5 and (D) Histatin 8, in 20 mM MOPSO, pH 7.0, at 25 °C. The top panels show the differential power signals (thermograms) recorded for each injection, whereas the bottom panels present the binding isotherm constructed by fitting the points corresponding to the integration of the peaks in the thermograms (representing the heat released during each injection) as a function of the metal/ Hst molar ratio.

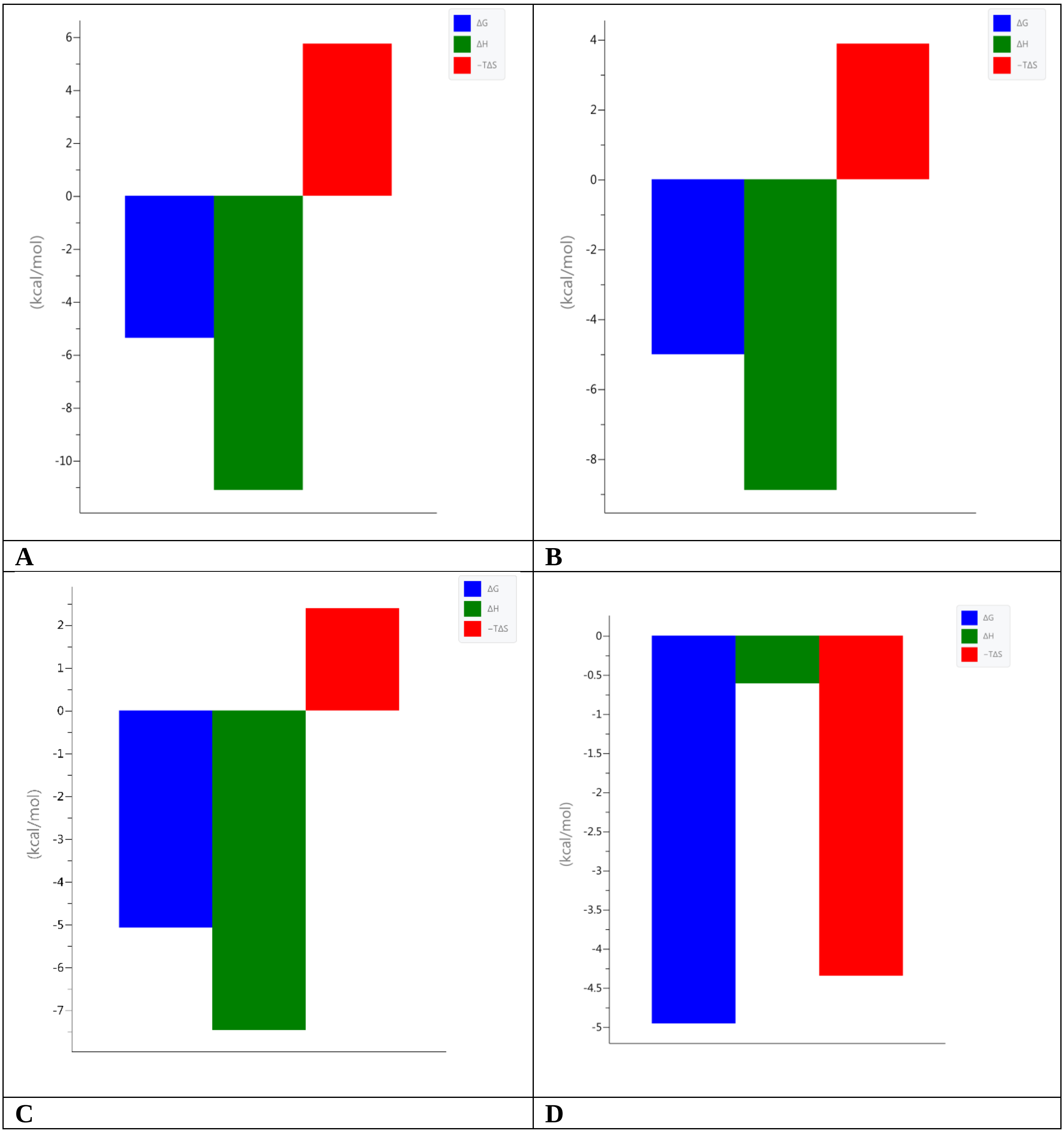

Figure S2. Signature plots of the titration of Ni(II) into (A) Histatin 5 and (B) Histatin 8 and Zn (II) into (C) Histatin 5 and (D) Histatin 8, in 20 mM MOPSO, pH 7.0, at 25 °C.

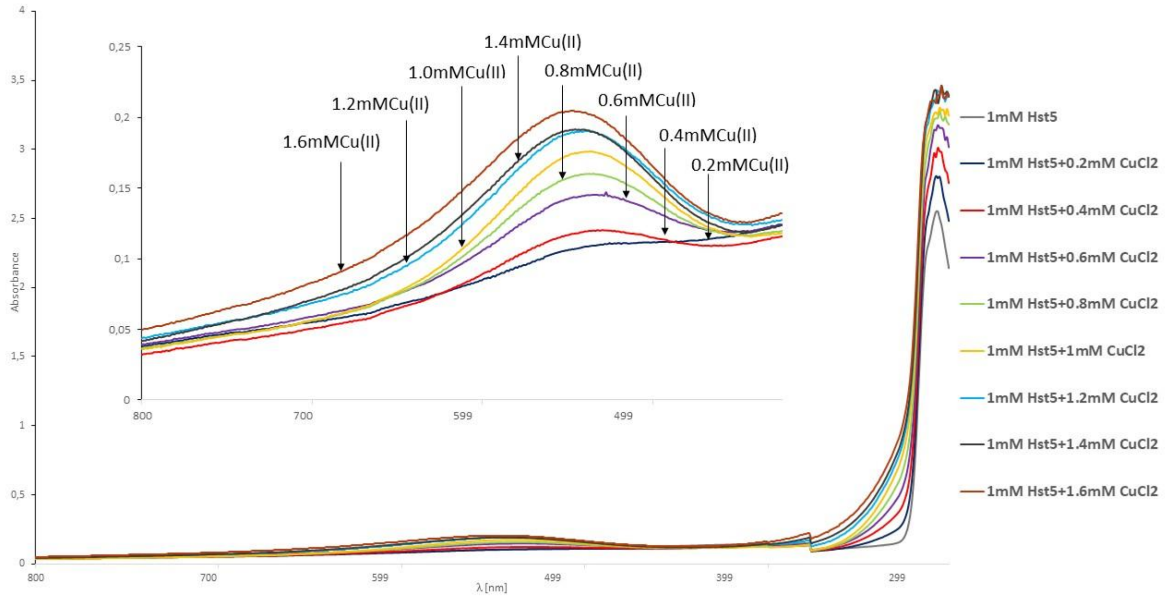

Figure S3. UV-ViS results of Cu(II)-Hst5 binding in 20 mM MOPSO, pH 7.0, at 25 °C.

The step at ~340 nm most likely results from the light source switching in the spectrophotometer, which does not affect the interpretation of the spectra in the discussed range.

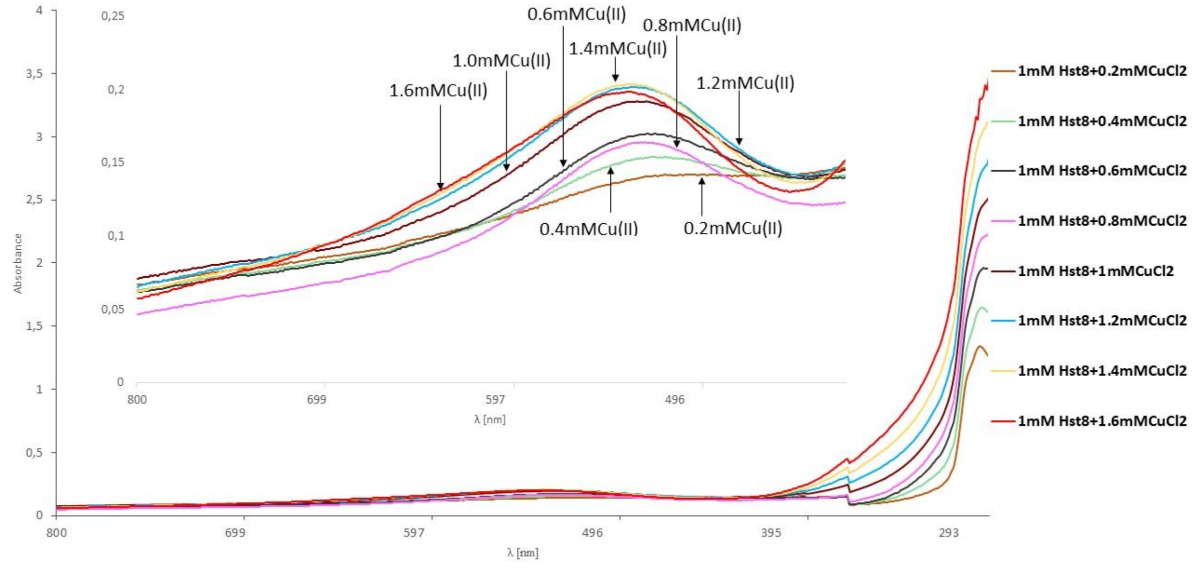

Figure S4. UV-ViS results of Cu(II)-Hst8 binding in 20 mM MOPSO, pH 7.0, at 25 °C. The step at ~340 nm most likely results from the light source switching in the spectrophotometer, which does not affect the interpretation of the spectra in the discussed range.

Table S2. Maximum wavelenght of absorbance in the d-d transition region for Cu(II) and Ni(II) complexes with Hst5 and Hst8 at a 1:1 ratio, measured in MOPSO buffer, at pH 7.0

	**λ_{max} [nm]**
**Cu(II)-Hst5**	520
**Cu(II)-Hst8**	515
**Ni(II)-Hst5**	420
**Ni(II)-Hst8**	420

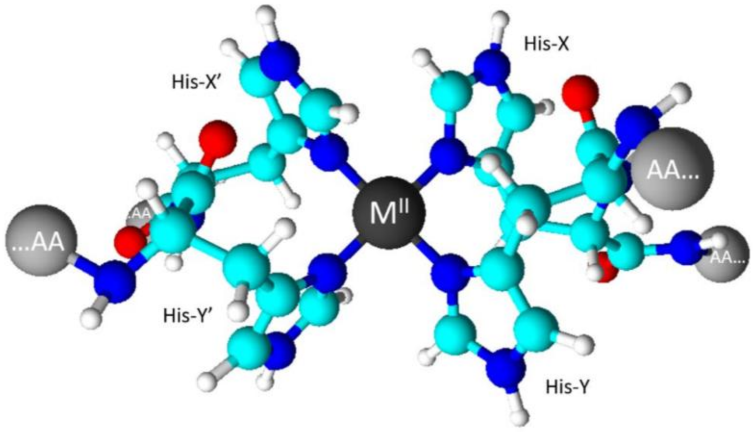

Figure S5. Proposed coordination mode of M(II) complexes with the interpeptide imidazoles, that can lead to dimer formation.
